# Supplementary material for: A Promising Thermodynamic Study of Hole Transport Materials to Develop Solar Cells: 1,3-Bis(N-carbazolyl)benzene and 1,4-Bis(diphenylamino)benzene
Source: Molecules. 2022 Jan 7;27(2):381. doi: 10.3390/molecules27020381 (PMC8779908; doi:10.3390/molecules27020381)
Supplement: Supplementary file 1 [file molecules-27-00381-s001.zip › molecules-1510708-supplementary.pdf]

A Promising Thermodynamic Study of Hole Transport Materials to Develop Solar Cells: 1,3-Bis(*N*-carbazolyl)benzene and 1,4-Bis(diphenylamino)benzene

Juan Mentado-Morales [1,*], Arturo Ximello-Hernández [2,*], Javier Salinas-Luna [3], Vera L. S. Freitas [4] and Maria D. M. C. Ribeiro da Silva [4]

[1] Instituto de Industrias, Universidad del Mar, Puerto Ángel, San Pedro Pochutla Oaxaca 70902, Mexico
[2] Procesos Bioalimentarios, Universidad Tecnológica de Tehuacán, Prolongación de la 1 Sur 1101, San Pablo Tepetzingo, Tehuacán Puebla 75859, Mexico
[3] Instituto de Ecología, Universidad del Mar, Puerto Ángel, San Pedro Pochutla Oaxaca 70902, Mexico; jsl@angel.umar.mx
[4] Centro de Investigação em Química da Universidade do Porto (CIQUP), Department of Chemistry and Biochemistry, Faculty of Science, University of Porto, Rua do Campo Alegre, P-4169-007 Porto, Portugal; vera.freitas@fc.up.pt (V.L.S.F.); mdsilva@fc.up.pt (M.D.M.C.R.d.S.)
* Correspondence: juan@angel.umar.mx (J.M.-M.); arturo.ximello@uttehuacan.edu.mx (A.X.-H.)

Supplementary Materials

TABLE OF CONTENTS

		Page
**A1.**	Spectroscopy	A3
**A2.**	Differential Scanning Calorimetry (DSC)	A10
**A3.**	Thermogravimetry	A16
**A4.**	Combustion Calorimetry	A23
**A5.**	Theoretical Calculations	A25

FIGURE INDEX

		Page
**Figure S1.**	^1H spectrum of NMR for NCB	A4
**Figure S2.**	^1H spectrum of NMR DAB	A5
**Figure S3.**	^{13}C NMR spectrum for NCB	A6
**Figure S4.**	^{13}C NMR spectrum for DAB	A7
**Figure S5.**	Raman spectrum for DAB.	A8
**Figure S6.**	Raman spectrum for NCB.	A9
**Figure S7.**	Heat capacity in crystalline phase of 1,3-Bis(*N*-carbazolyl)benzene (NCB)	A13
**Figure S8.**	Heat capacity in crystalline phase of 1,4-Bis(diphenylamino)benzene (DAB)	A15
**Figure S9.**	Representative mass-loss as a function of the temperature of the vaporisation process for NCB and DAB	A17
**Figure S10.**	Representative derivative curves (dm/dt) vs T of the vaporisation process for NCB and DAB	A17
**Figure S11.**	Dependence of $\ln((dm/dt)\cdot T)$ versus $1/T$ for NCB and DAB	A22

TABLE INDEX

		Page
**Table S1.**	Detailed results of spectroscopic data for NCB and DAB	A3
**Table S2.**	Physical properties and parameters of the compounds used in this work	A11
**Table S3.**	Heat capacity in the solid phase for NCB measured in the temperature range of (274.15–332.15) K at constant pressure obtained by differential scanning calorimetry	A12
**Table S4.**	Heat capacity in the solid phase for DAB measured in the temperature range of (274.15–332.15) K at constant pressure obtained by differential scanning calorimetry	A14

**Table S5.**	Representative experimental data for the determination of the vaporization enthalpy of 1,3-Bis(N-carbazolyl) benzene (NCB) in the temperature range of (550.0 to 650.0) K …………………	A18
**Table S6.**	Representative experimental data for the determination of the vaporization enthalpy of 1,4-Bis(diphenylamino) benzene (DAB) in the temperature range of (500.0 to 600.0) K………………	A19

**Table S7.**	Results of the Combustion Experiments for NCB at T = 298.15 K and $p°$ = 0.1 MPa	A23
**Table S8.**	Results of the Combustion Experiments for DAB at T = 298.15 K and $p°$ = 0.1 MPa	A24
**Table S9.**	G3(MP2)//B3LYP enthalpies, $H^{\circ}_{298.15\mathrm{K}}$, and experimental gas-phase standard ($p°$ = 0.1 MPa) molar enthalpies of formation, $\Delta_f H^{\circ}_m(\mathrm{g})$, at T = 298.15 K, for NCB and DAB and for the auxiliary species ………………………………………………………………………………………...	A25
	**References**	A26

A1. Spectroscopy

The study compounds were purified by recrystallization with 60% ethanol and 40% ethyl acetate mixture. After purification, the identity of the compounds was obtained by spectroscopy of nuclear and carbon-13 magnetic resonances, (^{1}H NMR), (^{13}C NMR) and RAMAN, respectively. The spectroscopic data for 1,3-Bis(*N*-carbazolyl)benzene (NCB) and for 1,4-Bis(diphenylamino)benzene (DAB) are found in Table S1. The proton spectra (^{1}H NMR) for NCB and DAB, are shown in Figures S1 and S2, respectively, while the carbon-13 spectra (^{13}C NMR) are found in Figures S3 and S4. Figures S5 and S6 are the Raman spectra corresponding to aromatic ring stretch and C-N stretch vibrations of the DAB and NCB, respectively. Raman spectra were acquired with a fiber-coupled EZ Raman-N spectrometer working in 785 nm as excitation wavelength and a pixel resolution of ~ 1.45 cm^{-1} per pixel.

Table S1. Detailed results of spectroscopic data for NCB and DAB.

Method	Spectroscopic Values
1,3-Bis(N-carbazolyl)benzene (NCB)	
^{1}H NMR (300 MHz, $CDCl_3$)	δ 7.28 (ddd, *J* = 8.1, 1.2 Hz, 4H), 7.41 (ddd, *J* = 8.4, 7.2, 1.5 Hz, 4H), 7.51 (dt, *J* = 8.1, 0.9 Hz, 4H), 7.63 (dd, *J* = 0.9, 1.8 Hz, 1H), 7.65 (dd, J = 2.1, 0.6 Hz, 1H), 7.75-7.80 (m, 2H), 8.11 (ddd, *J* = 7.8, 1.2, 0.9 Hz, 4H)
^{13}C NMR (75 MHz, $CDCl_3$)	δ 109.8, 120.6, 123.7, 125.4, 125.9, 126.3, 131.3, 139.5, 140.7.
RAMAN (50-2000 cm^{-1})	(300, 410, 426, 696, 748,1006, 1011,1240, 1314, 1338, 1456, 1490, 1578, 1596 1622) cm^{-1}.
1,4-Bis(diphenylamino)benzene (DAB)	
^{1}H NMR (300 MHz, $(CD_3)_2CO$)	δ 6.99-7.08 (m, 16H), 7.29 (m, 8H).
^{13}C NMR (75 MHz, $(CD_3)_2CO$)	δ 128.0, 129.0, 130.9, 134.7, 148.5, 153.3.
RAMAN (0-2000 cm^{-1})	(202, 258, 328, 370, 414, 552, 610, 734, 998, 1024, 1162, 1270,1314,1342, 1588, 1606) cm^{-1}.

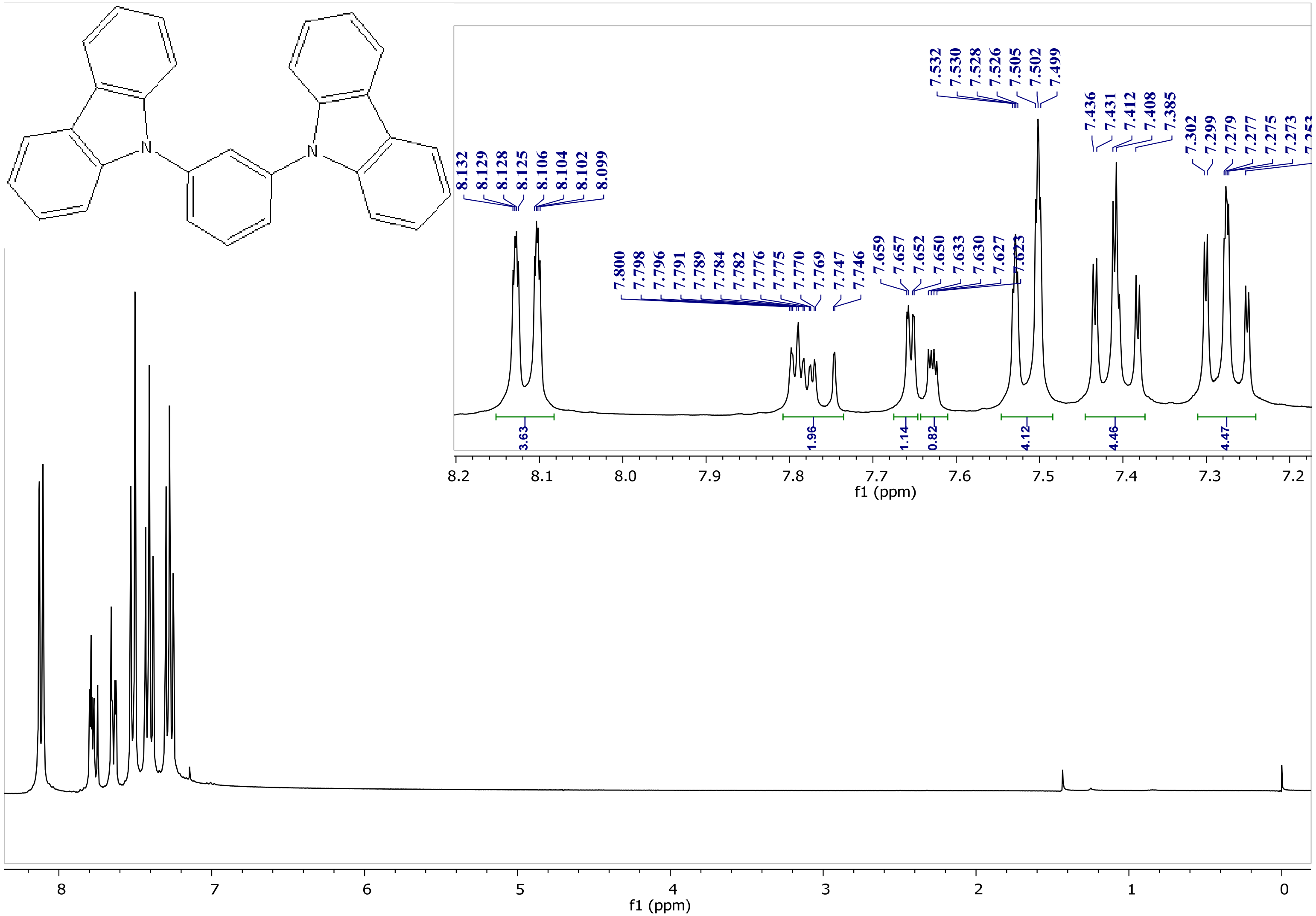

Figure S1. ^{1}H spectrum of NMR (300 MHz, CDCl3) for NCB and the integration of their peaks.

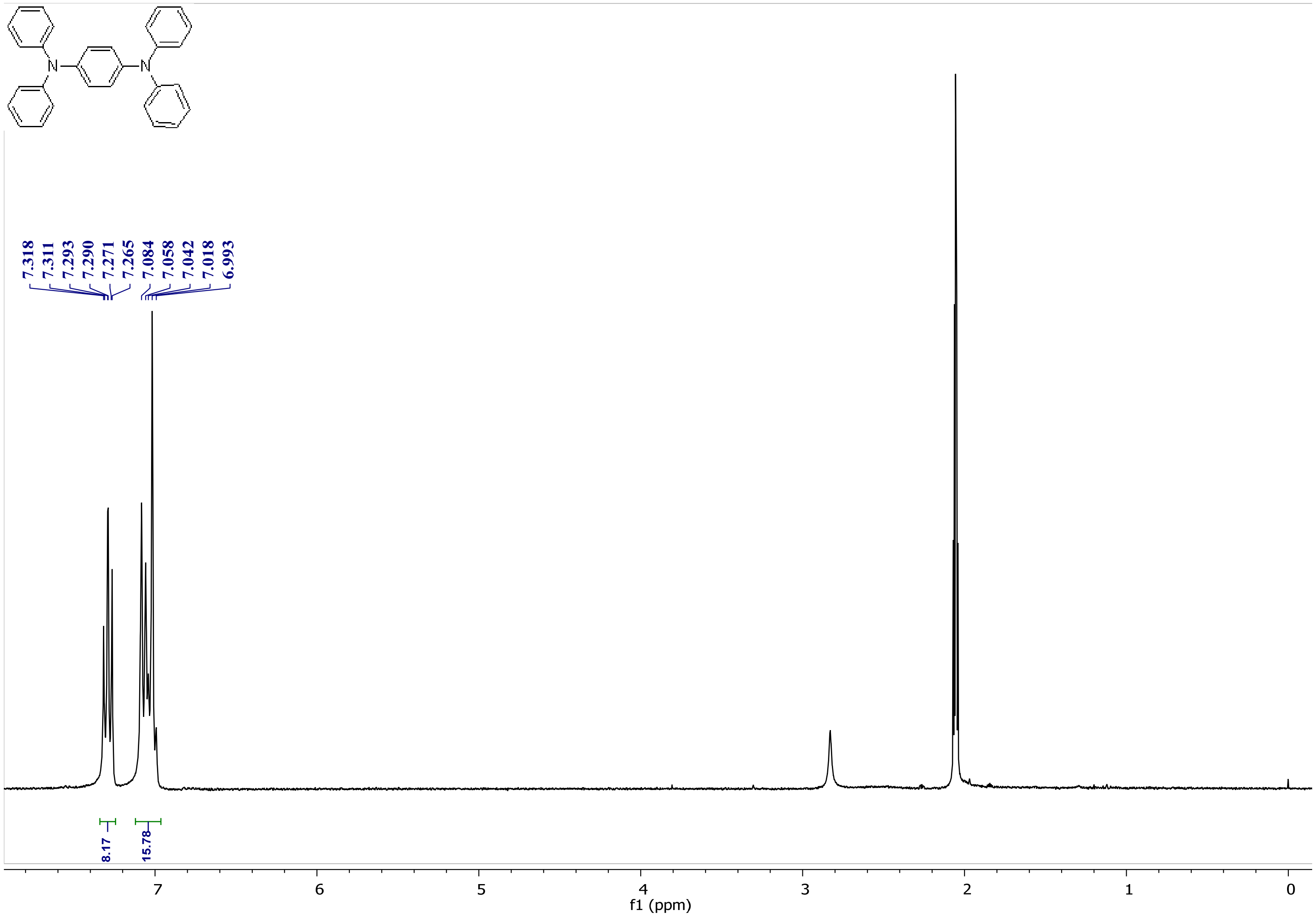

Figure S2. ^{1}H spectrum of NMR (500 MHz, CDCl3) for DAB and the integration of their peaks.

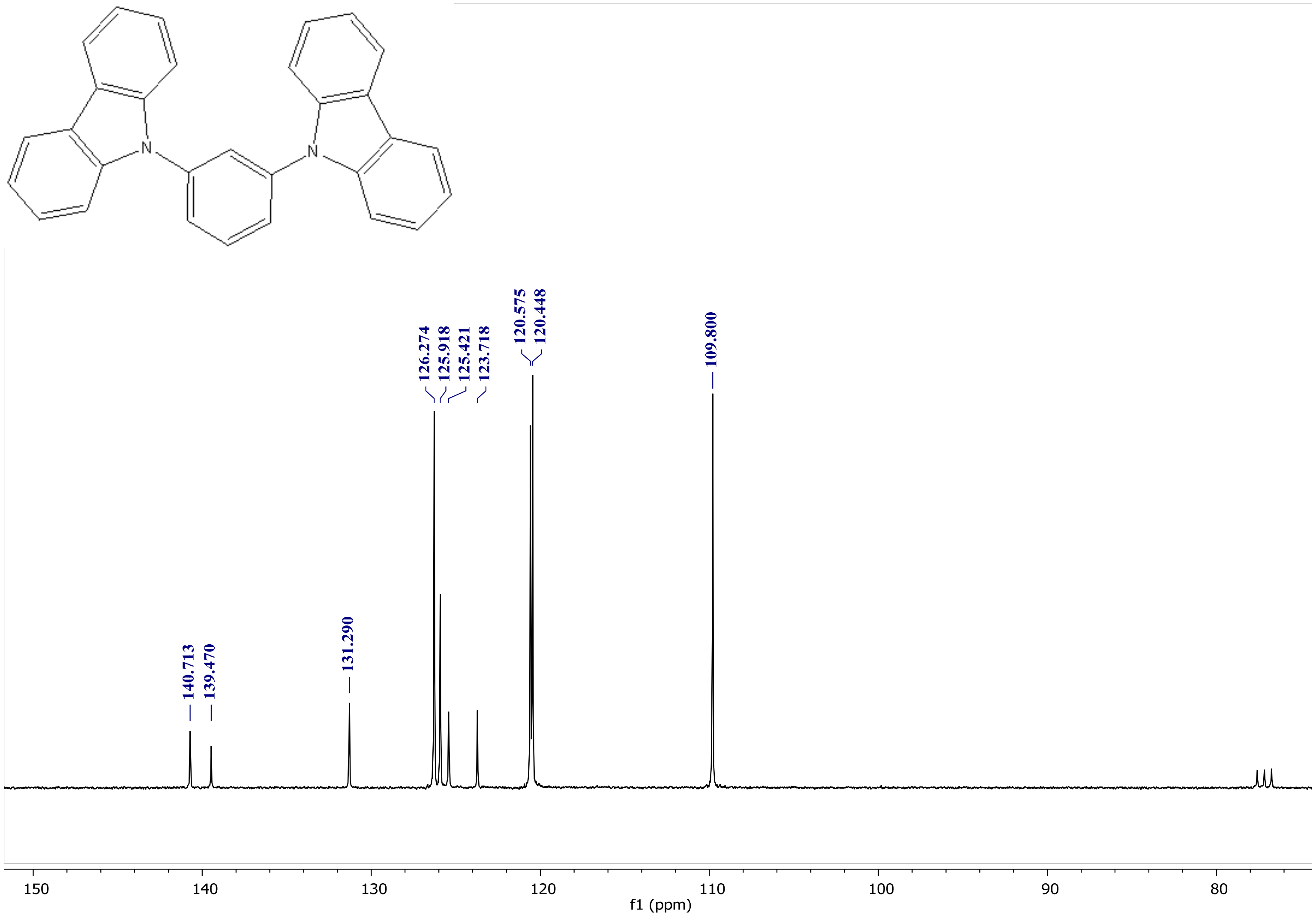

Figure S3. ^{13}C NMR spectrum (75 MHz, CDCl3) for NCB.

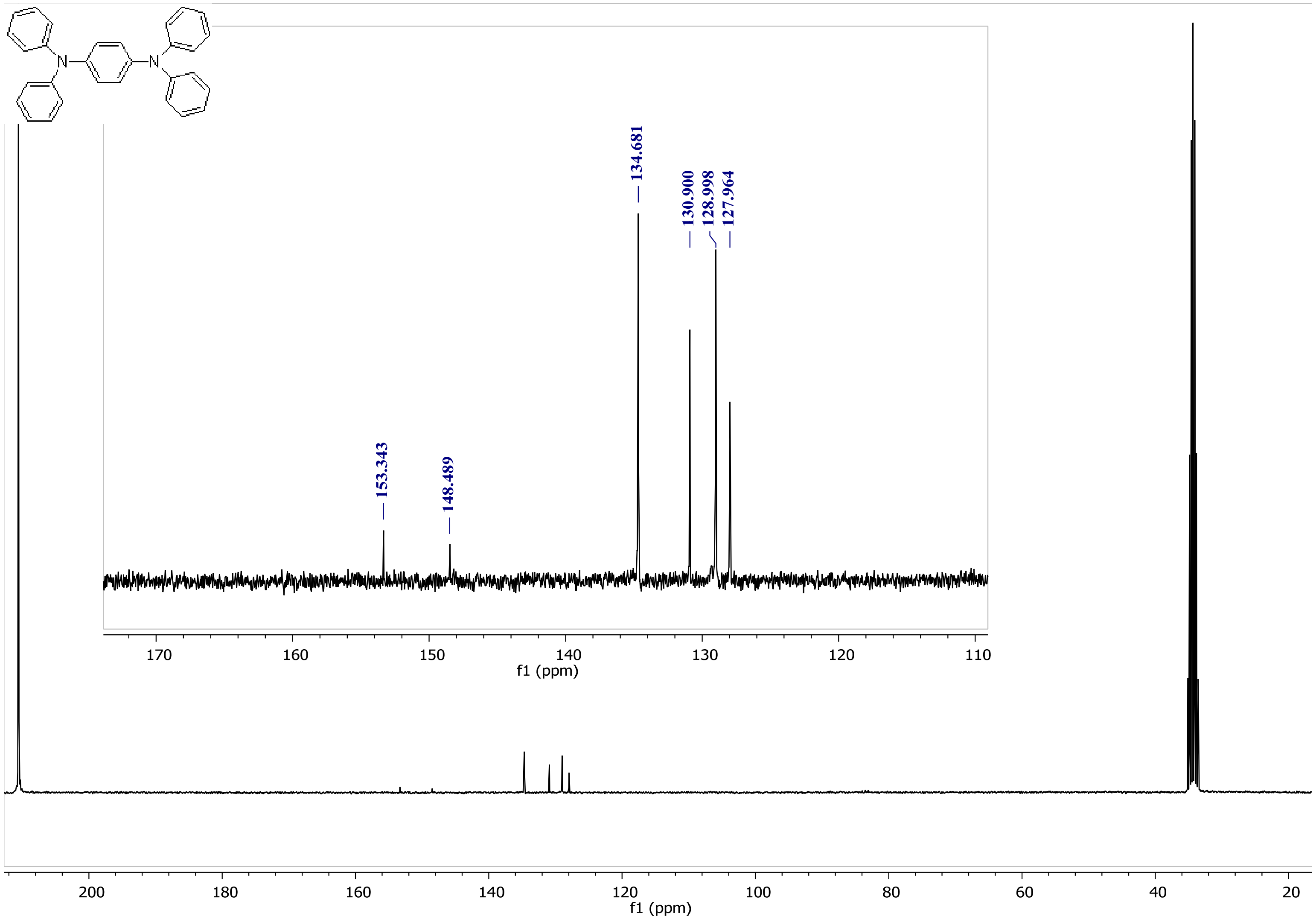

Figure S4. ^{13}C NMR spectrum (125 MHZ, CDCl3) for DAB.

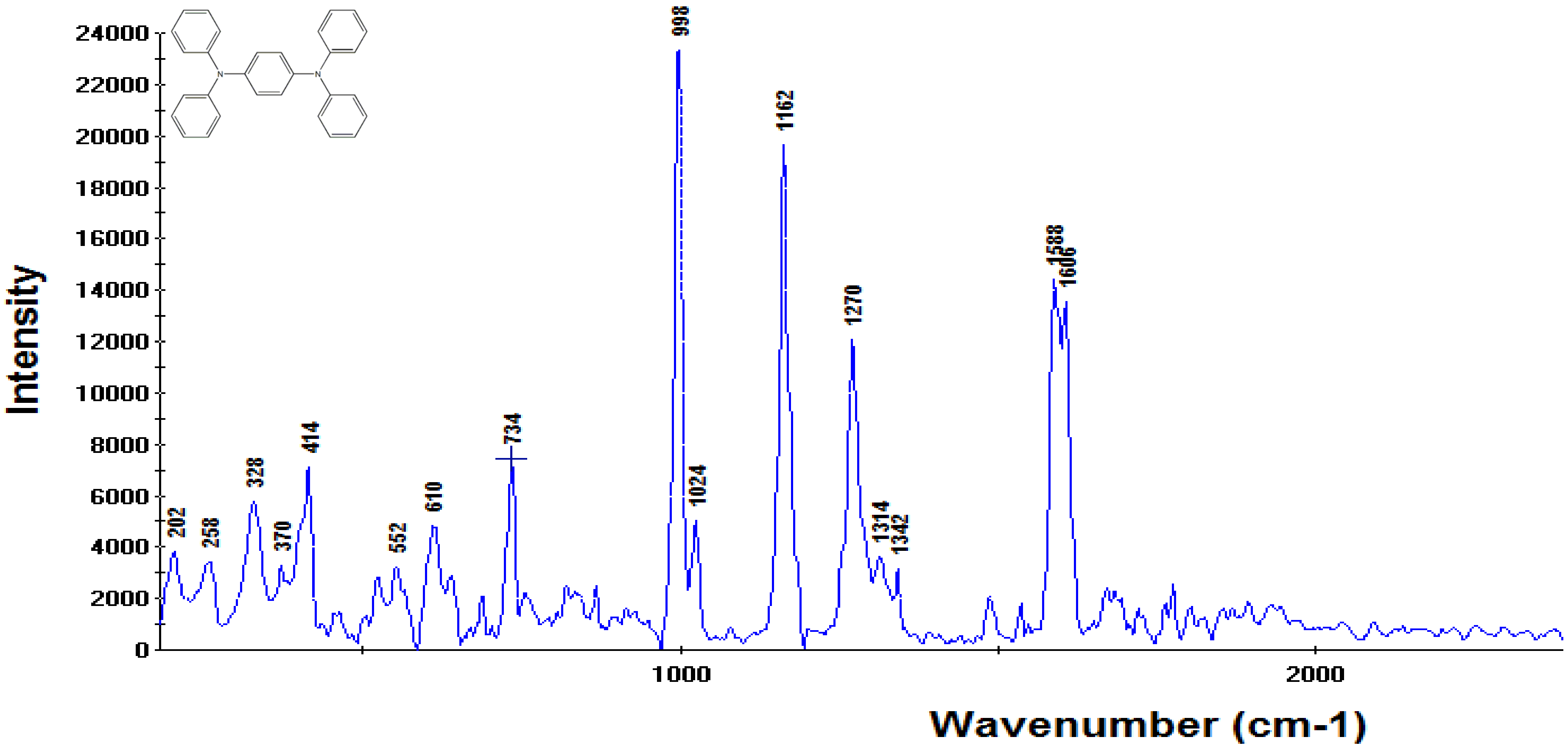

Figure S5. Raman spectrum for DAB.

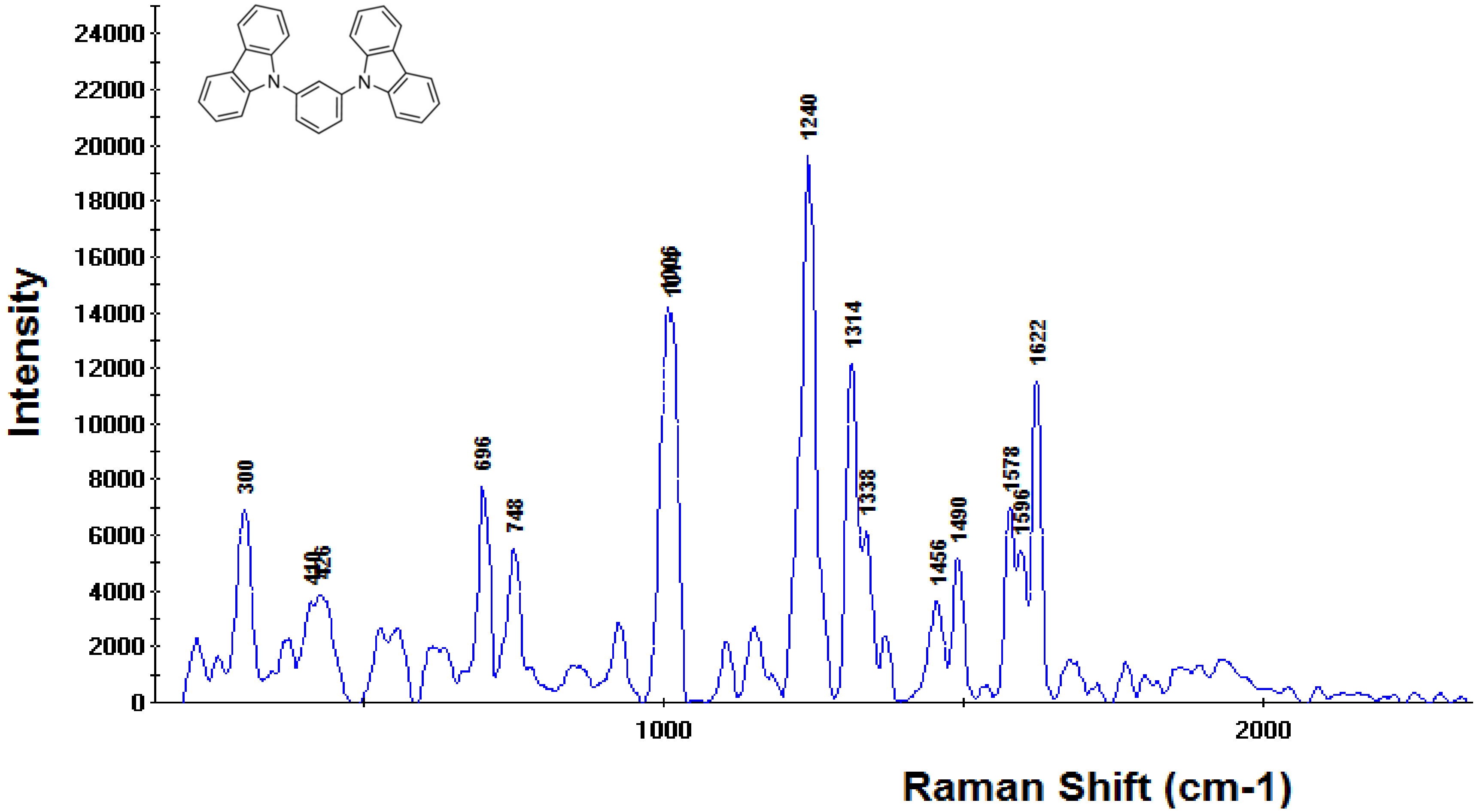

Figure S6. Raman spectrum for NCB.

A2. Differential Scanning Calorimetry (DSC)

After the purification, the purity of 1,3-Bis(*N*-carbazolyl)benzene (NCB) and 1,4-Bis(diphenylamino)benzene (DAB) was analysed by Differential Scanning Calorimetry (DSC) and using the van't Hoff equation, which relates the decrease in the melt temperature of the pure component to the amount of impurity involved [1]. The determinations were realized with a TA Instruments DSC Q2000 calorimeter, which was calibrated with the fusion of high-purity metallic indium. The masses used in all experiments were of ~ 3 mg, and a scanning rate of 5 $K \cdot min^{-1}$ and flow rate of 50 $ml \cdot min^{-1}$ of nitrogen were applied. The results of purity, melting temperature, and fusion enthalpy obtained by DSC are shown in Table S2. The uncertainties represent the standard deviation of the mean of a set of at least five measurements.

The crystalline molar heat capacities at constant pressure, $C_{\mathrm{p}}^{\mathrm{o}}(\mathrm{cr})$, for 1,3-Bis(*N*-carbazolyl)benzene (NCB) and for 1,4-Bis(diphenylamino)benzene (DAB) were determined with a Perkin-Elmer DSC 8000 calorimeter over the temperature range of (274.15 to 332.15) K using synthetic sapphire as reference and applying the two steps method [2,3]. The experimental values of heat capacities for NCB and for DAB, are given in Tables S3 and S4, respectively. Additionally, the $C_{\mathrm{p}}^{\mathrm{o}}(\mathrm{cr})$ as a function of the temperature, between 274.15 K and 332.15 K, of NCB and DAB, were adjusted to a second-degree polynomial function, expressed in eqs. A1 and A2, respectively. Graphs of the heat capacities of NCB and DAB are exposed in Figures S5 and S6, respectively.

Table S2. Physical properties and parameters of the compounds used in this work.

Compound	M/g·mol$^{-1,\ a}$	ρ/g·cm^{-3}	$-(\partial u/\partial p)_T$/J·g^{-1}·M	x^b	T_{fus}/K^b	$\Delta_{cr}^{l}H_m^o(T_{fus})$/kJ·mo	C_p^o(cr,l)/J·K^{-1}·mo
Paraffin oil	14.0270	0.860[b]	0.257[c]		—	—	31.10[4]
Cotton	28.1820	1.500[5]	0.289[6]		—	—	36.88[4]
NCB	408.4932	1.171[c]	0.2[5]	0.9996 ± 0.0003	450.84 ± 0.04	31.47 ± 0.11	445.16 ± 0.70[b]
DAB	412.5250	1.190[c]	0.2[5]	0.9997 ± 0.0001	475.72 ± 0.02	45.92 ± 0.20	458.94 ± 0.24[b]

[a]Molar mass are based on 2016 IUPAC recommendations.[7] [b]Experimental value. [c]Value estimated.

Table S3. Heat capacity in the solid phase for 1,3-Bis(N-carbazolyl)benzene (NCB) measured in the temperature range of (274.15–332.15) K at constant pressure obtained by differential scanning calorimetry.

T/K.	C_p^o(cr)/J·K^{-1}·mol^{-1}				
	Series				Mean value
	1	2	3	4	
274.15	399.10	400.32	401.55	401.55	400.63
275.15	402.37	401.96	404.00	402.77	402.78
276.15	403.59	404.00	405.23	405.23	404.51
277.15	404.82	405.23	406.04	406.45	405.64
278.15	406.86	407.27	408.49	408.49	407.78
279.15	408.90	409.72	408.90	410.13	409.41
280.15	410.13	410.13	410.94	411.35	410.64
281.15	412.99	412.99	413.80	414.62	413.60
282.15	414.62	414.62	415.85	415.85	415.24
283.15	416.66	416.66	416.25	417.89	416.87
284.15	417.48	417.07	417.89	418.71	417.79
285.15	419.93	419.52	420.75	421.16	420.34
286.15	423.20	423.61	424.42	424.02	423.81
287.15	424.02	423.61	424.42	424.83	424.22
288.15	425.65	424.02	424.83	426.47	425.24
289.15	428.10	427.28	427.69	428.51	427.90
290.15	430.14	429.33	430.14	429.73	429.84
291.15	431.78	431.37	431.37	432.19	431.68
292.15	434.23	432.59	433.41	433.82	433.51
293.15	434.23	434.23	434.64	434.23	434.33
294.15	437.50	436.68	436.68	436.68	436.89
295.15	439.13	439.54	439.95	440.36	439.75
296.15	441.99	441.99	442.40	443.22	442.40
297.15	443.62	443.62	444.03	444.44	443.93
**298.15**	**444.44**	**444.85**	**445.26**	**446.07**	**445.16**
299.15	445.67	445.67	445.26	446.48	445.77
300.15	447.30	446.48	447.71	448.93	447.61
301.15	449.34	449.75	450.16	450.16	449.85
302.15	450.57	450.98	450.98	451.79	451.08
303.15	452.61	452.61	452.61	453.43	452.82
304.15	453.84	453.43	454.24	454.65	454.04
305.15	455.88	455.88	456.29	455.88	455.98
306.15	456.70	457.10	457.51	457.51	457.21
307.15	457.92	458.74	458.74	458.74	458.54
308.15	459.96	459.96	460.78	460.37	460.27
309.15	461.60	462.01	462.82	461.60	462.01
310.15	463.23	462.82	463.64	463.23	463.23
311.15	463.23	463.23	464.05	464.05	463.64
312.15	466.09	466.50	466.91	466.91	466.60
313.15	466.91	467.32	467.72	467.32	467.32
314.15	467.72	468.95	469.36	469.77	468.95
315.15	469.36	469.77	471.81	470.58	470.38
316.15	470.58	471.40	472.63	471.81	471.61
317.15	471.81	473.04	473.44	473.04	472.83
318.15	472.63	475.49	476.71	474.67	474.88

319.15	474.26	475.49	475.89	475.89	475.38
320.15	475.08	476.71	477.12	477.12	476.51
321.15	476.71	477.53	478.75	477.94	477.73

**Table S3.** (Continuation).					
T/K	C_p(cr)/J·K^{-1}·mol^{-1}				Mean value
	Series				
	1	2	3	4	
322.15	477.53	479.16	480.80	479.98	479.37
323.15	478.35	480.39	480.39	479.98	479.78
324.15	479.57	481.20	482.02	482.02	481.20
325.15	480.80	482.84	483.66	482.84	482.54
326.15	482.02	484.06	484.47	484.88	483.86
327.15	483.25	484.47	485.70	485.29	484.68
328.15	484.88	486.52	486.92	486.92	486.31
329.15	485.29	486.92	488.15	487.74	487.03
330.15	486.52	487.74	488.97	489.37	488.15
331.15	487.33	488.56	489.78	490.19	488.97
332.15	489.78	490.60	492.23	491.83	491.11
$C_p^o(\mathrm{cr})/\left(\mathrm{J{\cdot}K^{-1}{\cdot}mol^{-1}}\right) = -0.0089(T/\mathrm{K})^2 + 6.948(T/\mathrm{K}) - 839.28$, ($r^2 = 0.9994$)					(SI1)

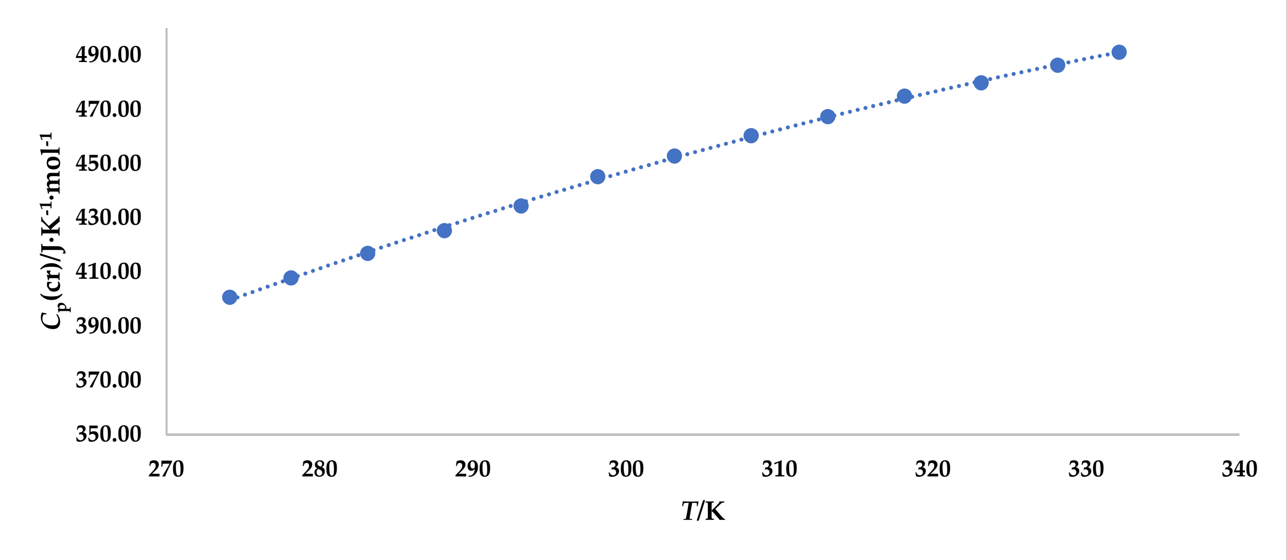

Figure S7. Heat capacity in crystalline phase of 1,3-Bis(N-carbazolyl)benzene (NCB).

Table S4. Heat capacity and equation in the solid phase for 1,4-Bis(diphenylamine)benzene (DAB) measured in the temperature range of (274.15–332.15) K at constant pressure obtained by differential scanning calorimetry.

T/K	$C_p(cr)/J{\cdot}K^{-1}{\cdot}mol^{-1}$				
	Series				Mean value
	1	2	3	4	
274.15	420.78	421.60	422.01	421.60	421.50
275.15	421.60	422.01	422.43	422.43	422.12
276.15	423.25	424.90	424.08	424.08	424.08
277.15	424.90	424.90	425.31	425.31	425.11
278.15	426.14	426.55	426.96	426.96	426.65
279.15	428.20	428.20	428.61	428.61	428.41
280.15	428.20	428.61	429.03	429.03	428.72
281.15	431.50	431.09	431.50	431.09	431.30
282.15	431.50	432.33	432.33	431.91	432.02
283.15	433.56	433.56	433.98	433.56	433.67
284.15	434.39	434.39	435.21	435.21	434.80
285.15	437.28	437.69	437.69	437.69	437.59
286.15	438.93	438.93	439.34	439.34	439.14
287.15	440.58	440.99	441.40	441.40	441.09
288.15	442.23	441.81	442.64	442.23	442.23
289.15	444.29	443.88	444.29	443.88	444.09
290.15	445.53	445.11	445.53	445.53	445.43
291.15	446.76	445.94	446.76	446.35	446.45
292.15	448.41	448.00	448.00	448.83	448.31
293.15	449.24	448.83	448.83	448.41	448.83
294.15	449.65	449.65	449.65	449.65	449.65
295.15	452.95	452.54	452.54	453.36	452.85
296.15	455.84	455.84	455.43	455.02	455.53
297.15	458.32	457.49	457.49	457.49	457.70
**298.15**	**459.14**	**458.73**	**458.73**	**459.14**	**458.94**
299.15	460.38	459.55	459.14	459.97	459.76
300.15	461.62	461.20	461.20	460.79	461.20
301.15	463.27	462.03	462.03	462.85	462.55
302.15	464.92	464.09	464.50	463.68	464.30
303.15	466.57	465.74	466.57	466.15	466.26
304.15	467.39	467.80	467.39	467.80	467.60
305.15	470.28	469.45	469.87	469.45	469.76
306.15	473.17	471.10	470.69	470.69	471.41
307.15	473.58	472.75	472.75	472.34	472.86
308.15	475.23	474.40	473.99	473.99	474.40
309.15	477.70	476.05	476.05	476.47	476.57
310.15	478.12	478.53	477.70	477.29	477.91
311.15	480.18	478.94	478.94	479.35	479.35
312.15	481.83	480.59	480.18	480.18	480.70
313.15	483.07	481.83	481.83	482.24	482.24
314.15	486.37	483.89	483.89	483.89	484.51
315.15	486.78	485.13	485.13	485.54	485.65
316.15	488.43	486.78	486.78	487.19	487.30
317.15	490.08	487.19	488.02	488.43	488.43
318.15	491.73	490.49	490.49	490.90	490.90
319.15	494.20	492.55	492.97	492.55	493.07

320.15	495.44	494.20	494.20	494.20	494.51
321.15	497.09	495.44	496.27	496.27	496.27

Table S4. (Continuation).

T/K	C_p(cr)/J·K^{-1}·mol^{-1}				Mean value
	Series				
	1	2	3	4	
323.15	499.16	498.33	497.92	498.33	498.44
324.15	501.22	500.39	500.39	500.39	500.60
325.15	502.87	502.46	502.04	502.46	502.46
326.15	504.52	504.11	503.69	503.28	503.90
327.15	505.34	505.34	504.93	505.34	505.24
328.15	507.82	507.41	506.99	507.41	507.41
329.15	509.06	509.06	509.06	509.06	509.06
330.15	510.29	510.71	510.29	510.71	510.50
331.15	511.94	511.94	512.36	511.94	512.05
332.15	513.18	514.01	512.36	513.59	513.29

$$C_p^o(\mathrm{cr})/\left(\mathrm{J\cdot K^{-1}\cdot mol^{-1}}\right) = 0.0012(T/\mathrm{K})^2 + 0.9022(T/\mathrm{K}) + 84.565,\ (r^2 = 0.9996) \quad \text{(SI2)}$$

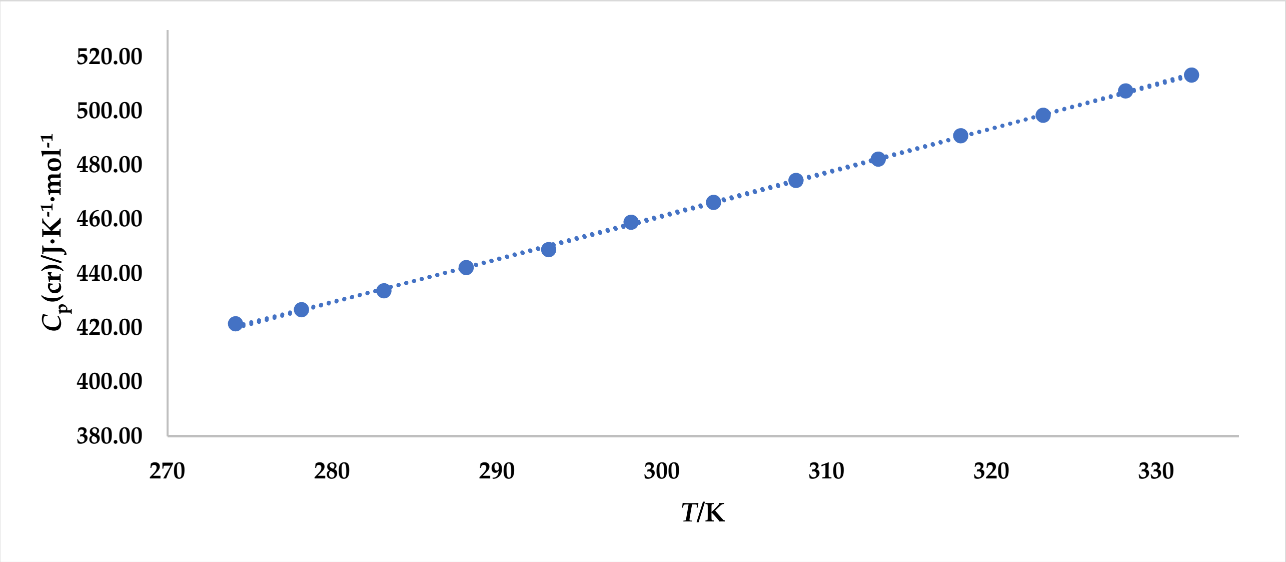

Figure S8. Heat capacity in crystalline phase of 1,4-Bis(diphenylamino)benzene (DAB).

A3. Thermogravimetry

The thermogravimetric experiments were carried out on a TA Instruments TGA Q500. This equipment has a thermobalance with a temperature and mass sensitivities of ±0.1 K and ±0.1 μg, respectively. The device was calibrated for mass with NIST reference masses into a range of (100 to 1000) mg. Additionally, for the temperature calibration of the instrument an Alumel-Nickel alloy with Curie Point Temperature values of 425.75 K and 631.35 K, respectively, was used. In the experiments, masses of 16.6 mg for NCB and 10.5 mg for DAB were utilized and heating rate of 10.0 $K \cdot min^{-1}$ with a flow rate of nitrogen of 100 $cm^3 \cdot min^{-1}$, were applied. Representative mass loss and mass loss rate as a function of the temperature for NCB and DAB, respectively, in Figures S9 and S10, are shown. Tables S5 and S6 shows experimental data for the determination of the vaporization enthalpy of 1,3-Bis(*N*-carbazolyl)benzene (NCB) and 1,4-Bis(diphenylamine)benzene (DAB). The fitted equations, the correlations coefficients, r^2, and the uncertainties for the y-intercept, σ_a, and the slope, σ_b, of each experimental series are also given. The weighted average values of the vaporization enthalpies and their uncertainties were calculated applying the equations suggested by Bevington and Robinson [8]. Finally, Figure S11 shows the dependence of $\ln((dm/dt) \cdot T)$ as a function of the temperature ($1/T$) of all experimental series, derived from the vaporization experiments of each study compounds.

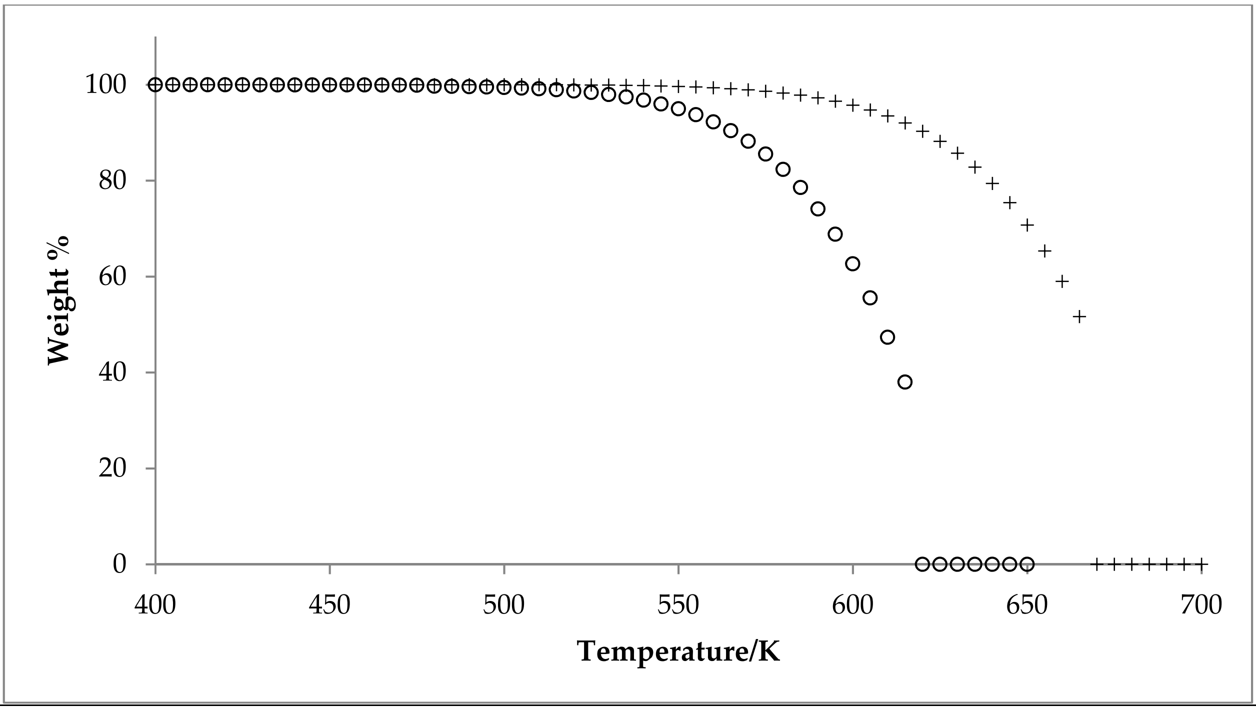

Figure S9. Representative mass-loss as a function of the temperature of the vaporisation process for NCB and DAB. 1,3-Bis(N-carbazolyl)benzene (NCB) (+); 1,4-Bis (diphenylamino)benzene (DAB) (○).

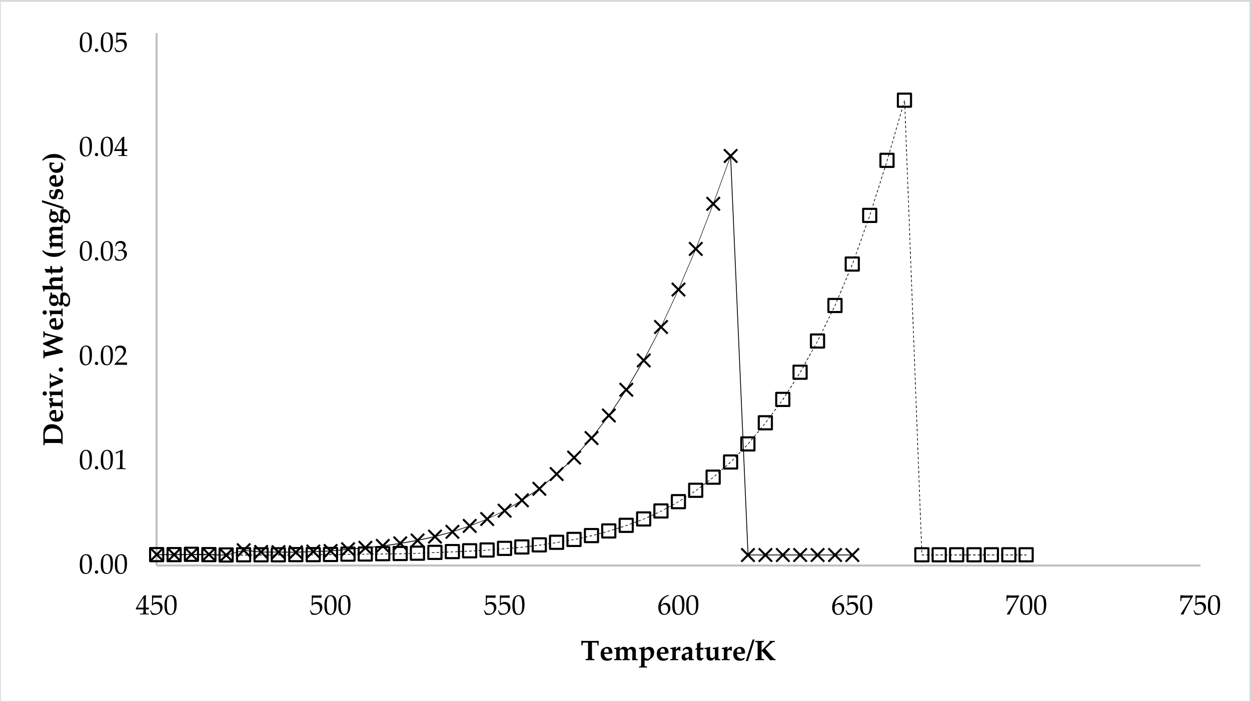

Figure S10. Representative derivative curves (d*m*/d*t*) vs *T* of the vaporisation process for NCB and DAB. - - - - trendline for 1,3-Bis(N-carbazolyl)benzene (NCB) (□); —— trendline for 1,4-Bis(diphenylamine)benzene (DAB) (×).

Table S5. Representative experimental data for the determination of the vaporization enthalpy of 1,3-Bis(N-carbazolyl)benzene (NCB) in the temperature range of (550.0 to 650.0) K, which were determined by using a TA Instruments Q500 device.

T/K	m/mg	$(dm/dt)\cdot10^9$/kg·s^{-1}	$(1/T)\cdot10^3$/K^{-1}	$-\ln[(dm/dt)\cdot T]$
Series 1				
550.00	16.6151	0.6021	1.818	14.921
555.00	16.5950	0.7384	1.802	14.708
560.00	16.5695	0.9424	1.786	14.455
565.00	16.5376	1.1899	1.770	14.213
570.00	16.4976	1.4724	1.754	13.991
575.00	16.4478	1.8404	1.739	13.759
580.00	16.3861	2.2805	1.724	13.536
585.00	16.3098	2.8146	1.709	13.317
590.00	16.2163	3.4351	1.695	13.109
595.00	16.1018	4.1887	1.681	12.902
600.00	15.9623	5.0904	1.667	12.699
605.00	15.7934	6.1669	1.653	12.499
610.00	15.5897	7.4318	1.639	12.304
615.00	15.3450	8.8927	1.626	12.116
620.00	15.0535	10.6169	1.613	11.931
625.00	14.7053	12.6390	1.600	11.749
630.00	14.2936	14.8808	1.587	11.577
635.00	13.8080	17.4926	1.575	11.408
640.00	13.2393	20.4814	1.563	11.242
645.00	12.5737	23.8926	1.550	11.080
650.00	11.7970	27.8601	1.538	10.919
$\ln((dm/dt)\cdot T) = 11.3–14396.9/T$; $r^2 = 0.9997$; $\sigma_a = 0.10$; $\sigma_b = 58.5$; $\Delta_l^g H_m^o$(600.0 K)/kJ·mol^{-1} = 119.7 ± 0.5				
Series 2				
550.00	14.0298	0.6240	1.818	-14.885
555.00	14.0088	0.7705	1.802	-14.665
560.00	13.9822	0.9931	1.786	-14.402
565.00	13.9495	1.2068	1.770	-14.198
570.00	13.9086	1.5040	1.754	-13.969
575.00	13.8583	1.8751	1.739	-13.740
580.00	13.7957	2.3052	1.724	-13.525
585.00	13.7193	2.8228	1.709	-13.314
590.00	13.6253	3.4305	1.695	-13.110
595.00	13.5107	4.1959	1.681	-12.901
600.00	13.3721	5.0713	1.667	-12.703
605.00	13.2040	6.1281	1.653	-12.505
610.00	13.0019	7.3586	1.639	-12.314
615.00	12.7595	8.8413	1.626	-12.122
620.00	12.4699	10.5548	1.613	-11.937
625.00	12.1264	12.4457	1.600	-11.764
630.00	11.7195	14.7070	1.587	-11.589
635.00	11.2398	17.2690	1.575	-11.421
640.00	10.6782	20.1923	1.563	-11.256
645.00	10.0207	23.5940	1.550	-11.093
650.00	9.2482	27.7567	1.538	-10.923
$\ln((dm/dt)\cdot T) = 10.9–14198.1/T$; $r^2 = 0.9998$; $\sigma_a = 0.07$; $\sigma_b = 42.2$; $\Delta_l^g H_m^o$(600.0 K)/kJ·mol^{-1} = 118.0 ± 0.4				

Table S5. (Continuation).				
Series 3				
550.00	13.8033	0.6023	1.818	-14.920
555.00	13.7824	0.7857	1.802	-14.645
560.00	13.7566	0.9366	1.786	-14.461
565.00	13.7242	1.2008	1.770	-14.203
570.00	13.6841	1.4938	1.754	-13.976
575.00	13.6348	1.8183	1.739	-13.771
580.00	13.5733	2.2613	1.724	-13.544
585.00	13.4980	2.7932	1.709	-13.324
590.00	13.4049	3.4264	1.695	-13.112
595.00	13.2912	4.2020	1.681	-12.899
600.00	13.1528	5.0824	1.667	-12.701
605.00	12.9850	6.1322	1.653	-12.504
610.00	12.7825	7.3341	1.639	-12.317
615.00	12.5393	8.8097	1.626	-12.126
620.00	12.2496	10.5514	1.613	-11.937
625.00	11.9034	12.5698	1.600	-11.754
630.00	11.4951	14.8488	1.587	-11.580
635.00	11.0134	17.2837	1.575	-11.420
640.00	10.4514	20.2639	1.563	-11.253
645.00	9.7958	23.5329	1.550	-11.096
650.00	9.0353	27.1727	1.538	-10.944
$\ln((dm/dt)\cdot T) = 11.1–14281.6/T$; $r^2 = 0.9997$; $\sigma_a = 0.10$; $\sigma_b = 59.3$; $\Delta_l^g H_m^o$(600.0 K)/kJ·mol^{-1} = 118.7 ± 0.5				
Series 4				
550.00	18.8595	0.6535	1.818	-14.839
555.00	18.8375	0.8155	1.802	-14.608
560.00	18.8099	0.9954	1.786	-14.400
565.00	18.7754	1.2898	1.770	-14.132
570.00	18.7323	1.6008	1.754	-13.907
575.00	18.6792	1.9235	1.739	-13.715
580.00	18.6131	2.4373	1.724	-13.469
585.00	18.5320	2.8979	1.709	-13.288
590.00	18.4321	3.6467	1.695	-13.049
595.00	18.3104	4.4417	1.681	-12.844
600.00	18.1634	5.4028	1.667	-12.639
605.00	17.9846	6.5421	1.653	-12.440
610.00	17.7706	7.8818	1.639	-12.245
615.00	17.5140	9.3393	1.626	-12.067
620.00	17.2083	11.0699	1.613	-11.889
625.00	16.8435	13.1693	1.600	-11.708
630.00	16.4133	15.5396	1.587	-11.534
635.00	15.9057	18.2437	1.575	-11.366
640.00	15.3124	21.3646	1.563	-11.200
645.00	14.6179	24.9088	1.550	-11.039
650.00	13.8127	28.8390	1.538	-10.885
$\ln((dm/dt)\cdot T) = 11.1–14240.9/T$; $r^2 = 0.9997$; $\sigma_a = 0.10$; $\sigma_b = 59.2$; $\Delta_l^g H_m^o$(600.0 K)/kJ·mol^{-1} = 118.4 ± 0.5				
Weighted average value: <$\Delta_l^g H_m^o$(NCB, 600 K)>/kJ·mol^{-1} = 118.6 ± 0.4				

Parameters σ_a and σ_b represents the standard deviation of the intercept and slope of the function $\ln((dm/dt)\cdot T)$ vs $1/T$. The uncertainty for each vaporisation enthalpy value was computed as $\sigma_b\cdot R\cdot 10^{-3}$. The weighted average value μ and its

standard deviation σ, were calculated as $\mu = \sum_i^N (x_i/\sigma_i^2) / \sum_i^N (1/\sigma_i^2)$ and $\sigma = (N/\sum_i^N (1/\sigma_i^2))^{1/2}$, where x_i and σ_i are the experimental data of each N vaporization enthalpy and its respective uncertainty [8].

Table S6. Representative experimental data for the determination of the vaporization enthalpy of 1,4-Bis(diphenylamino)benzene (DAB) in the temperature range of (500.0 to 600.0) K, which were determined by using a TA Instruments Q500 device.

T/K	m/mg	$(dm/dt)\cdot 10^9$/kg·s^{-1}	$(1/T)\cdot 10^3$/K^{-1}	$-\ln[(dm/dt)\cdot T]$
Series 1				
500.00	10.6641	0.4411	2.000	-15.327
505.00	10.6492	0.5658	1.980	-15.068
510.00	10.6296	0.7364	1.961	-14.795
515.00	10.6044	0.9464	1.942	-14.534
520.00	10.5724	1.1944	1.923	-14.292
525.00	10.5325	1.4796	1.905	-14.068
530.00	10.4828	1.8358	1.887	-13.843
535.00	10.4210	2.2892	1.869	-13.613
540.00	10.3441	2.8443	1.852	-13.386
545.00	10.2492	3.5138	1.835	-13.166
550.00	10.1321	4.2965	1.818	-12.956
555.00	9.9888	5.2672	1.802	-12.743
560.00	9.8140	6.4049	1.786	-12.538
565.00	9.6021	7.7353	1.770	-12.341
570.00	9.3460	9.3120	1.754	-12.146
575.00	9.0390	11.1517	1.739	-11.957
580.00	8.6720	13.2946	1.724	-11.773
585.00	8.2371	15.7318	1.709	-11.596
590.00	7.7230	18.5333	1.695	-11.424
595.00	7.1203	21.6737	1.681	-11.259
600.00	6.4172	25.1040	1.667	-11.103
$\ln((dm/dt)\cdot T) = 10.1–12705.0/T$; $r^2 = 0.9997$; $\sigma_a = 0.09$; $\sigma_b = 51.7$; $\Delta_l^g H_m^o$(550.0 K)/kJ·mol^{-1} = 105.6 ± 0.4				
Series 2				
500.00	11.4736	0.3797	2.000	-15.477
505.00	11.4603	0.5420	1.980	-15.111
510.00	11.4422	0.6889	1.961	-14.862
515.00	11.4196	0.8649	1.942	-14.624
520.00	11.3900	1.1090	1.923	-14.366
525.00	11.3524	1.3895	1.905	-14.131
530.00	11.3049	1.7627	1.887	-13.884
535.00	11.2456	2.2125	1.869	-13.647
540.00	11.1706	2.7789	1.852	-13.410
545.00	11.0773	3.4427	1.835	-13.186
550.00	10.9620	4.2401	1.818	-12.969
555.00	10.8205	5.2390	1.802	-12.748
560.00	10.6464	6.3480	1.786	-12.547
565.00	10.4348	7.7531	1.770	-12.338
570.00	10.1793	9.3317	1.754	-12.144
575.00	9.8715	11.2130	1.739	-11.952
580.00	9.5033	13.3567	1.724	-11.768
585.00	9.0667	15.8256	1.709	-11.590
590.00	8.5492	18.6371	1.695	-11.418
595.00	7.9412	21.8479	1.681	-11.251
600.00	7.2312	25.4262	1.667	-11.091
$\ln((dm/dt)\cdot T)$. = $10.7–13059.5/T$; $r^2 = 0.9993$; $\sigma_a = 0.15$; $\sigma_b = 80.0$; $\Delta_l^g H_m^o$(550.0 K)/kJ·mol^{-1} = 108.6 ± 0.7				

**Table S6.** (Continuation).				
Series 3				
500.00	10.2745	0.3960	2.000	-15.435
505.00	10.2606	0.5154	1.980	-15.162
510.00	10.2424	0.6810	1.961	-14.873
515.00	10.2189	0.8582	1.942	-14.632
520.00	10.1898	1.1018	1.923	-14.372
525.00	10.1518	1.4099	1.905	-14.116
530.00	10.1045	1.7493	1.887	-13.891
535.00	10.0449	2.2164	1.869	-13.645
540.00	9.9703	2.7650	1.852	-13.415
545.00	9.8773	3.4113	1.835	-13.195
550.00	9.7622	4.2422	1.818	-12.968
555.00	9.6203	5.2145	1.802	-12.753
560.00	9.4466	6.3662	1.786	-12.544
565.00	9.2354	7.7151	1.770	-12.343
570.00	8.9799	9.3221	1.754	-12.145
575.00	8.6729	11.1682	1.739	-11.956
580.00	8.3063	13.3015	1.724	-11.772
585.00	7.8712	15.7587	1.709	-11.594
590.00	7.3576	18.5143	1.695	-11.425
595.00	6.7564	21.5801	1.681	-11.263
600.00	6.0567	25.0257	1.667	-11.106
$\ln((\mathrm{d}m/\mathrm{d}t)\cdot T) = 10.7–13042.7/T$; $r^2 = 0.9993$; $\sigma_a = 0.14$; $\sigma_b = 77.1$; $\Delta_l^g H_m^o(550.0\ \mathrm{K})/\mathrm{kJ\cdot mol^{-1}} = 108.4 \pm 0.6$				
Series 4				
500.00	12.2890	0.3980	2.000	-15.430
505.00	12.2747	0.5417	1.980	-15.112
510.00	12.2562	0.6967	1.961	-14.850
515.00	12.2322	0.8993	1.942	-14.585
520.00	12.2019	1.1445	1.923	-14.334
525.00	12.1633	1.4487	1.905	-14.089
530.00	12.1143	1.8287	1.887	-13.847
535.00	12.0525	2.2852	1.869	-13.615
540.00	11.9751	2.8713	1.852	-13.377
545.00	11.8785	3.5754	1.835	-13.148
550.00	11.7590	4.4091	1.818	-12.930
555.00	11.6119	5.4330	1.802	-12.712
560.00	11.4319	6.6050	1.786	-12.507
565.00	11.2133	7.9894	1.770	-12.308
570.00	10.9492	9.6252	1.754	-12.113
575.00	10.6324	11.5134	1.739	-11.925
580.00	10.2541	13.7045	1.724	-11.743
585.00	9.8051	16.2202	1.709	-11.565
590.00	9.2753	19.0952	1.695	-11.394
595.00	8.6535	22.3476	1.681	-11.228
600.00	7.9277	26.0051	1.667	-11.068
$\ln((\mathrm{d}m/\mathrm{d}t)\cdot T) = 10.8–13049.3/T$; $r^2 = 0.9992$; $\sigma_a = 0.15$; $\sigma_b = 84.3$; $\Delta_l^g H_m^o(550.0\ \mathrm{K})/\mathrm{kJ\cdot mol^{-1}} = 108.5 \pm 0.7$				
Weighted average value: $\langle\Delta_l^g H_m^o(\mathrm{NCB},\ 550\ \mathrm{K})\rangle/\mathrm{kJ\cdot mol^{-1}} = 107.2 \pm 0.6$				

Parameters σ_a and σ_b represents the standard deviation of the intercept and slope of the function $\ln((\mathrm{d}m/\mathrm{d}t)\cdot T)$vs $1/T$. The uncertainty for each vaporisation enthalpy value was computed as $\sigma_b\cdot R\cdot 10^{-3}$. The weighted average value μ and its

standard deviation σ, were calculated as $\mu = \sum_i^N (x_i/\sigma_i^2)/\sum_i^N (1/\sigma_i^2)$ and $\sigma = (N/\sum_i^N (1/\sigma_i^2))^{1/2}$, where x_i and σ_i are the experimental data of each N vaporization enthalpy and its respective uncertainty [8].

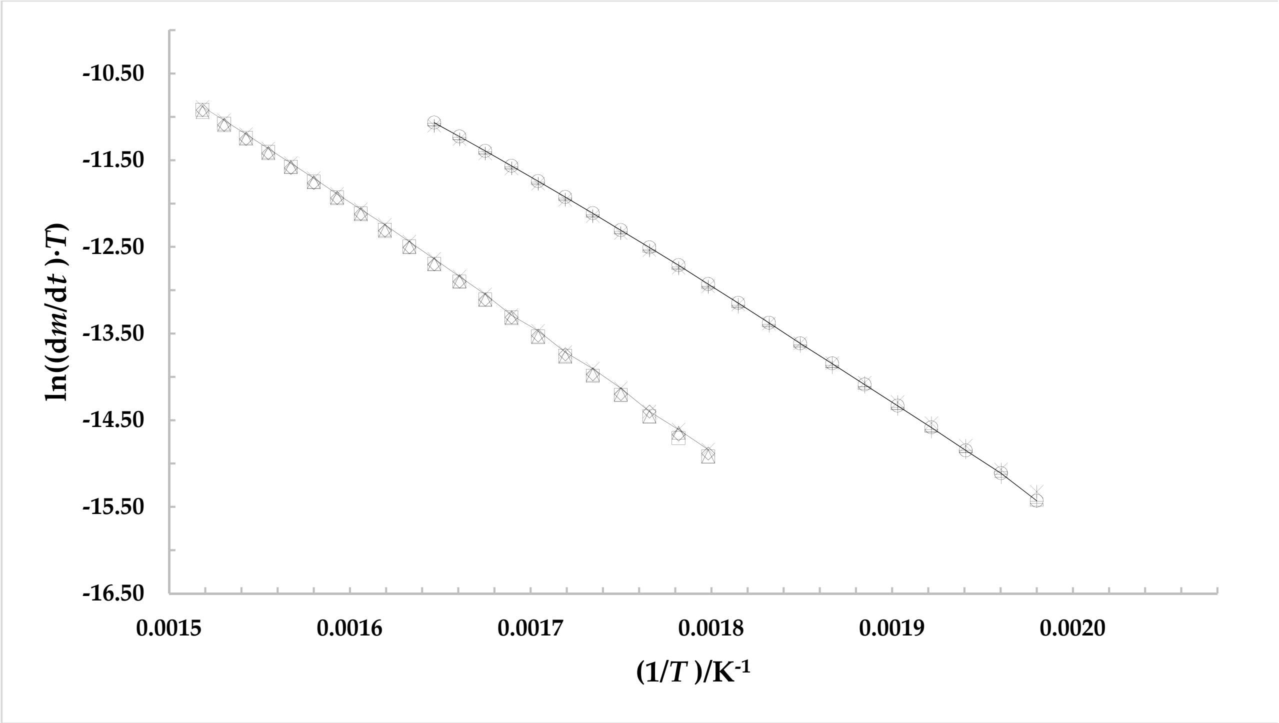

Figure S11. Dependence of ln((d*m*/d*t*)·*T*) versus 1/*T* for NCB and DAB. □ NCB series 1, ◇ NCB series 2, △ NCB series 3, × NCB series 4; **+** DAB series 1, – DAB series 2, ▲ DAB series 3; ○ DAB series 4.

A4. Combustion Calorimetry

Results for the combustion experiments and the individual values of $\Delta_c u^o$(cr) for NCB and DAB compounds are given in Tables S7 and S8, respectively

Table S7. Results of the Combustion Experiments for NCB at T = 298.15 K and $p°$ = 0.1 MPa.[a.]

Experiment	1	2	3	4	5	6	7
m'(NCB)/g	0.01031	0.01211	0.01150	0.01101	0.01177	0.01119	0.01166
m''(paraffin)/g	0.00277	0.00168	0.00314	0.00309	0.00315	0.00367	0.00282
m'''(cotton)/g	0.00055	0.00062	0.00054	0.00055	0.00053	0.00050	0.00050
m(Pt)/g	0.20265	0.02027	0.20268	0.20198	0.20235	0.20163	0.20252
T_i/K	296.4052	296.4589	296.1709	296.1832	296.4905	296.2753	296.2047
T_f/K	296.6631	296.7315	296.4598	296.4632	296.7896	296.5690	296.4883
ΔT_c/K	0.2559	0.2636	0.2857	0.2758	0.2907	0.2905	0.2809
ε_i(cont.)/J·K^{-1}	1.1078	1.0850	1.1091	1.1085	1.1093	1.1085	1.1091
ε_f(cont.)/J·K^{-1}	1.1057	1.0830	1.1091	1.1077	1.1096	1.1090	1.1087
-ΔU_{IBP}/J	518.6903	534.5867	579.6497	559.4609	589.8874	592.4209	569.8416
ΔU(HNO_3)/J	0.0758	0.0698	0.0758	0.0607	0.0638	0.0668	0.0668
(ΔU_{ign})/J	4.1840	4.1840	4.1840	4.1840	4.1840	4.1840	4.1840
ΔU_Σ/J	0.1982	0.2459	0.2235	0.2128	0.2296	0.2138	0.2282
-m''($\Delta_c u°$) (paraffin)/J	130.3953	79.0845	147.8127	145.4590	148.2834	172.7619	132.7490
-m'''($\Delta_c u°$) (cotton)/J	9.3199	10.5060	9.1504	9.3199	8.9810	8.4726	8.4726
(-$\Delta_c u°$) (NCB)/J·g^{-1}	36731.41	36720.38	36729.36	36730.94	36731.71	36720.72	36734.43
$\langle \Delta_c u^o \rangle$ = – (36728.4 ± 2.1) J·g^{-1},[b]							

[a]m'(NCB), mass of 1,3-Bis(N-carbazolyl)benzene; m''(paraffin), mass of paraffin oil; m'''(cotton), mass of the cotton thread; m(Pt) , mass of platinum which includes crucible and wire for ignition; T_i and T_f initial and final temperatures, respectively; ΔT_c, corrected temperature rise; ε_i(cont.) and ε_f(cont.), energies equivalent of the bomb contents before and after ignition, respectively; ΔU_{IBP}, energy of the isothermal bomb process; ΔU(HNO_3), energy of formation of nitric acid; ΔU_{ign}, ignition energy;; ΔU_Σ, state standard correction; $\Delta_c u^o$, standard massic energy of combustion.
[b]Uncertainty corresponds to the standard deviation of the mean for seven experiments.

Table S8. Results of the Combustion Experiments for DAB at T = 298.15 K and $p°$ = 0.1 MPa.[a]

Experiment	1	2	3	4	5	6	7
m'(DAB)/g	0.01174	0.01301	0.01383	0.01085	0.01260	0.01180	0.01187
m''(paraffin)/g	0.00439	0.00395	0.00359	0.00406	0.00540	0.00368	0.00425
m'''(cotton)/g	0.00060	0.00059	0.00065	0.00056	0.00057	0.00045	0.00062
m(Pt) /g	0.20258	0.20264	0.20234	0.20230	0.20214	0.20205	0.20236
T_i /K	296.2732	296.3399	296.3822	296.1807	296.3404	296.1839	296.6264
T_f /K	296.6032	296.6826	296.7329	296.4876	296.7114	296.5005	296.9593
ΔT_c /K	0.3256	0.3388	0.3463	0.3011	0.3645	0.3090	0.3259
ε_i(cont)/J·K^{-1}	1.1103	1.1117	1.1127	1.1092	1.1111	1.1100	1.1104
ε_f (cont)/J·K^{-1}	1.1142	1.1169	1.1186	1.1113	1.1177	1.1129	1.1144
-ΔU_{IBP}/J	661.0947	688.2091	703.4166	611.1921	740.6704	627.3410	659.7514
$\Delta U(HNO_3)$ /J	0.0877	0.1188	0.0979	0.0985	0.0835	0.1229	0.1188
(ΔU_{ign})/J	4.1840	4.1840	4.1840	4.1840	4.1840	4.1840	4.1840
ΔU_Σ/J	0.2151	0.2446	0.2643	0.1964	0.2304	0.2174	0.2191
-$m''(\Delta_c u°)$ (paraffin)/J	206.6553	185.9427	168.9960	191.1208	254.2001	173.2327	200.0649
-$m'''(\Delta_c u°)$ (cotton)/J	10.1671	9.9977	11.0144	9.4893	9.6588	7.6253	10.5060
(-$\Delta_c u°$) (DAB)/J·g^{-1}	37816.42	37809.85	37819.77	37814.29	37817.24	37808.62	37813.07
$\langle \Delta_c u° \rangle$ = – (37814.2 ± 1.5) J·g^{-1}[b]							

[a]The symbols have the same meaning as in Table S7. m'(DAB), mass of 1,4-Bis(diphenylamino)benzene.
[b]Uncertainty corresponds to the standard deviation of the mean for seven experiments.

A5. Theoretical Calculations

Table S9. G3(MP2)//B3LYP enthalpies, $H^{\circ}_{298.15\mathrm{K}}$, and experimental gas-phase standard (p° = 0.1 MPa) molar enthalpies of formation, $\Delta_f H^{\circ}_m(g)$, at T = 298.15 K, for NCB and DAB and for the auxiliary species. 1 a. u. (Hartree) corresponds to 2625.50 kJ·mol^{-1}.

Compound	Molecular structure	$H^{\circ}_{298.15\mathrm{K}}$ / a. u.	$\Delta_f H^{\circ}_m(g)$ / kJ·mol^{-1}
benzene	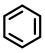	–231.835164	82.6 ± 0.7 [9]
1,3-Bis(*N*-carbazolyl)benzene	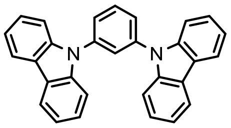	–1262.707262	This Study
1,4-Bis(diphenylamino)benzene	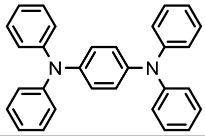	–1265.027939	This study
carbazole	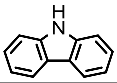	–516.611310	205.0 ± 3.0 [10]
diphenylamine	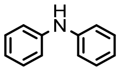	–517.772926	217.7 ± 1.8 [11]
hydrogen	H_2	–1.167343	
9-phenylcarbazole	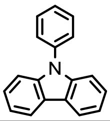	–747.271032	316.1 ± 9.4 [9]
triphenylamine	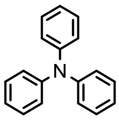	–748.431606	327.0 ± 4.2 [12]

REFERENCES

1. Brown, M. E. Determination of purity by differential scanning calorimetry (DSC). *J. Chem. Educ.* **1979**, *56*, 310–313.
2. Höhne, G. W. H.; Hemminger, W. F.; Flammersheim, H. -J. *Differential Scanning Calorimetry*, 2nd ed.; Springer-Verlag, New York, **2003**.
3. Sabbah, R.; Xu-wu, A.; Chickos, J. S.; Leitão, M. L. P.; Roux, M. V.; Torres, L. A. *Reference materials for calorimetry and differential thermal analysis*. Thermochim. Acta **1999**, *331*, 93–204.
4. Flores, H.; Amador, P. Standard molar enthalpies of formation of crystalline stereoisomers of aldono-1,4-lactones. *J. Chem. Thermodyn*. **2004**, *36*, 1019–1024.
5. Washburn, E. W. Standard states for bomb calorimetry. *Bur. Stand. J. Res*. **1933**, *10*, 525–558.
6. Good, W. D.; Lacina, J. L.; McCullough, J. P. Sulfuric Acid: Heat of Formation of Aqueous Solutions by Rotating-bomb Calorimetry. *J. Am. Chem. Soc*. **1960**, *82*, 5589–5591.
7. Meija, J.; Coplen, T. B.; Berglund, M.; Brand, W. A.; De Bièvre, P.; Gröning, M.; Holden, N. E.; Irrgeher, J.; Loss, R. D.; Walczyk, T.; Prohaska, T. Atomic weights of the elements 2013 (IUPAC Technical Report). *Pure Appl. Chem*. **2016**, *88*, 265–291.
8. Bevington, P. R.; Robinson, D. K. Data Reduction and Error Analysis for the Physical Sciences; McGraw-Hill: New York, 2003.
9. Pedley J. B. *Thermochemical data and structures of organic compounds*. College Station, Thermodynamics Research Centre: Texas, USA, 1994; Volume. 1.
10. Jimenez, P.; Roux, M.V.; Turrion, C., Thermochemical properties of N-heterocyclic compounds. III. Enthalpies of combustion, vapour pressures and enthalpies of sublimation, and enthalpies of formation of 9H-carbazole, 9-methylcarbazole, and 9-ethylcarbazole, *J. Chem. Thermodyn.*, **1990**, *22*, 721-726.
11. Freitas, V.L.S.; Gomes, J.R.B.; Liebman, J.F., Ribeiro da Silva, M.D.M.C. Energetic and reactivity properties of 9,10-dihydroacridine and diphenylamine: a comparative overview. *J. Chem. Thermodyn*. **2017**, *115*, 276-284.
12. Steele, W.V., The standard enthalpies of formation of the triphenyl compounds of the Group V elements.

1. Triphenylamine and the Ph-N bond-dissociation energy, *J. Chem. Thermodyn.*, **1978**, *10*, 441-444.
